# Pooled prevalence and determinants of antenatal care visits in countries with high maternal mortality: A multi-country analysis

**DOI:** 10.3389/fpubh.2023.1035759

**Published:** 2023-01-30

**Authors:** Dagmawi Chilot, Daniel Gashaneh Belay, Tigist Andargie Ferede, Kegnie Shitu, Melaku Hunie Asratie, Sintayehu Ambachew, Yadelew Yimer Shibabaw, Demiss Mulatu Geberu, Melkamu Deresse, Adugnaw Zeleke Alem

**Affiliations:** ^1^Center for Innovative Drug Development and Therapeutic Trials for Africa (CDT-Africa), College of Health Sciences, Addis Ababa University, Addis Ababa, Ethiopia; ^2^Department of Human Physiology, College of Medicine and Health Science, School of Medicine, University of Gondar, Gondar, Ethiopia; ^3^Department of Human Anatomy, College of Medicine and Health Science, School of Medicine, University of Gondar, Gondar, Ethiopia; ^4^Department of Epidemiology and Biostatistics, College of Medicine and Health Science, Institute of Public Health, University of Gondar, Gondar, Ethiopia; ^5^Department of Health Education and Behavioral Sciences, Institute of Public Health, College of Medicine and Health Sciences, University of Gondar, Gondar, Ethiopia; ^6^Department of Women and Family Health, College of Medicine and Health Science, School of Midwifery, University of Gondar, Gondar, Ethiopia; ^7^Department of Clinical Chemistry, School of Biomedical and Laboratory, University of Gondar, Gondar, Ethiopia; ^8^Department of Biochemistry, College of Medicine and Health Science, School of Medicine, University of Gondar, Gondar, Ethiopia; ^9^Department of Health Systems and Policy, College of Medicine and Health Science, Institute of Public Health, University of Gondar, Gondar, Ethiopia; ^10^Department of Physiotherapy, St. Peter's Specialized Hospital, Addis Ababa, Ethiopia

**Keywords:** antenatal care, women, maternal mortality, low- and middle-income countries, reproductive-age women

## Abstract

**Background:**

Complications during pregnancy and childbirth are the leading causes of maternal and child deaths and disabilities, particularly in low- and middle-income countries. Timely and frequent antenatal care prevents these burdens by promoting existing disease treatments, vaccination, iron supplementation, and HIV counseling and testing during pregnancy. Many factors could contribute to optimal ANC utilization remaining below targets in countries with high maternal mortality. This study aimed to assess the prevalence and determinants of optimal ANC utilization by using nationally representative surveys of countries with high maternal mortality.

**Methods:**

Secondary data analysis was done using recent Demographic and Health Surveys (DHS) data of 27 countries with high maternal mortality. The multilevel binary logistic regression model was fitted to identify significantly associated factors. Variables were extracted from the individual record (IR) files of from each of the 27 countries. Adjusted odds ratios (AOR) with a 95% confidence interval (CI) and *p*-value of ≤0.05 in the multivariable model were used to declare significant factors associated with optimal ANC utilization.

**Result:**

The pooled prevalence of optimal ANC utilization in countries with high maternal mortality was 55.66% (95% CI: 47.48–63.85). Several determinants at the individual and community level were significantly associated with optimal ANC utilization. Mothers aged 25–34 years, mothers aged 35–49 years, mothers who had formal education, working mothers, women who are married, had media access, households of middle-wealth quintile, richest household, history of pregnancy termination, female household head, and high community education were positively associated with optimal ANC visits in countries with high maternal mortality, whereas being rural residents, unwanted pregnancy, having birth order 2–5, and birth order >5 were negatively associated.

**Conclusion and recommendations:**

Optimal ANC utilization in countries with high maternal mortality was relatively low. Both individual-level factors and community-level factors were significantly associated with ANC utilization. Policymakers, stakeholders, and health professionals should give special attention and intervene by targeting rural residents, uneducated mothers, economically poor women, and other significant factors this study revealed.

## Background

Maternal and child mortality remains a substantial public health concern worldwide. However, the risk of these problems is even higher in low- and middle-income countries (1, 2). The United Nations (UN) came up with the Millennium Development Goals (MDGs) to reduce maternal and child mortality in the world community to be achieved by 2015 (3). Despite the country's significant progress in achieving the MDG-5, it has been limited and uneven (4, 5). How the goals were designed, the lack of stakeholder commitment and interest, scarce resources, and lack of accountability were some of the factors for the slow and unequal progress (6, 7).

Complications during pregnancy and childbirth are the leading causes of maternal and child deaths and disabilities (8). The World Health Organization (WHO) recommended antenatal care (ANC) visits as a key strategy to endorse pregnant women's health (9, 10). The organization previously recommended a minimum of four ANC visits throughout the pregnancy; however, it revised its recommended minimum number of ANC visits from four to eight contacts in 2016 to have a safe pregnancy and healthy baby (11). Timely and frequent ANC promotes existing disease treatments, vaccination, malaria prophylaxis, iron supplementation, nutrition counseling, HIV counseling and testing, and urinary tract infection treatment (12–14).

The lowest levels of antenatal care are witnessed in sub-Saharan Africa and South Asia. Individual-level and community-level factors were consistently reported as the most important influences for ANC utilization. In recent studies conducted in low- and middle-income countries, women's education, residence, wealth index, husband's education, mass media, marital status, women's autonomy, husband support, and healthcare accessibility were the commonly reported determinants (15–20). In addition, it is been criticized that the MDG targets were not designed based on enough evidence of feasibility in low-income countries (21, 22). Moreover, limitations in the MDG development process, structure, content, implementation, and enforcement were one of the key obstacles (4, 23–25).

Numerous studies focusing on determinants of antenatal care use in low- and middle-income countries have been conducted and identified important factors. However, a minimum of four ANC visits (ANC4+) utilization in countries with high maternal mortality was not addressed, and getting reliable data on the implementation of MDG and interpretation of progress reports were frequently reported challenges. Identifying gaps in ANC4+ use specifically in these countries is important for stakeholders including policy planners and program managers to increase the utilization of services that decreases maternal–child mortality. In addition, giving a panoramic view of the problem and detecting possible determinates in high maternal mortality countries could help to implement SDG3. Therefore, the objective of our study was to assess the prevalence of ANC4+ visits among women aged 15–49 years and the potential factors associated with it in countries with high maternal mortality. Our study will provide evidence-based recommendations to improve ANC utilization in those reproductive-age women on a large scale.

## Materials and methods

### Study design and setting

The Demography and Health Surveys employed a cross-sectional study design to collect the data. In this study, we only included countries with high maternal mortality and have publically available DHS data (26).

### Data source

This study is a secondary data analysis using the DHS data conducted in 27 countries. The DHS is a nationally representative survey that is conducted in low- and middle-income countries globally. We used individual record (IR) files to extract the study participants of this study. We weighted the sample using the individual weight of women (v005) to produce the proper representation. Hence, sample weights were generated by dividing (v005) by 1,000,000, and the total weighted sample size from the pooled data was 209,538 (Table 1).

**Table 1:** Maternal mortality, category, and year of the survey by the country.

**Country**	**Year of DHS survey**	**Maternal mortality/ 100,000**	**Category**
Afghanistan	2018/19	638	Very high
Benin	2017/18	397	High
Burkina Faso	2010	320	High
Burundi	2016/17	548	Very high
Cameroon	2018	529	Very high
Chad	2014/15	1,140	Extremely high
Congo	2011/12	378	High
Côte d'Ivoire	2011/12	617	Very high
Democratic Republic of the Congo	2013/14	473	High
Eswatini	2006/7	437	High
Ethiopia	2016	401	High
Gambia	2019/20	597	Very high
Ghana	2014	308	High
Guinea	2018	576	Very high
Haiti	2017/18	480	High
Kenya	2014	342	High
Lesotho	2014	544	Very high
Liberia	2019/20	661	Very high
Madagascar	2021	335	High
Malawi	2015/16	349	High
Mali	2018	562	Very high
Mauritania	2019/21	766	Very high
Sierra Leone	2019	1,120	Extremely high
Tanzania	2015/16	524	Very high
Togo	2013/14	396	High
Uganda	2016	375	High
Zimbabwe	2015	458	High

High, 300–499; Very high, 500–999; Extremely high, >1,000.

### Population

Women aged 15–49 years with a birth in the last 5 years receiving antenatal care from a skilled provider for the most recent birth were the study population. Sample weight was used to correct for over- and under-sampling and generalizability of the findings.

### Definition of variables

#### Outcome variable

Antenatal care visit was the outcome variable for this study. We dichotomized the ANC visits as inadequate and adequate according to the WHO classification (11). Inadequate ANC is <4 visits, whereas optimal if women had four and more visits.

#### Independent variables

Potential explanatory variables associated with completing optimal ANC visits were considered on two levels. Variables such as mother's age, maternal educational status, parity, marital status, sex of the household head, birth order, and wealth index were used at the individual level, whereas residence, community-level education, community-level poverty, and community-level media exposure were used as community-level variables.

### Operational definitions

Community-level media usage is the proportion of women in the community who use radio, TV, and newsletter, and it was categorized as low community-level media usage and high community-level media usage. “Low” refers to communities in which <50% of respondents had media access, while “high” indicates communities in which ≥50% of respondents had media access.

Community-level women's education refers to the proportion of women in the community who have formal education. It was categorized as low if communities in which < 50% of respondents had formal education and high if ≥50% of respondents had attended formal education.

Community-level poverty refers to the proportion of women in the community who had low-wealth quintiles. It was categorized as low if the proportion of low-wealth quintile households was < 50% and high if the proportion was ≥50%.

### Statistical analyses

STATA version 14.2 was used to clean, recode, and analyze the data. A multilevel binary logistic regression model was fitted to identify significantly associated factors. Both community- and individual-level variables with a *p*-value of ≤0.2 in the bi-variable analysis were included in the multivariable model. Adjusted OR (AOR) with 95% CI and *p* < 0.05 were applied to determine significantly associated factors.

### Model building and parameter estimation

Four models were applied, comprising the null model (model 0) containing no variables, which is used to check the variability of ANC visits in the community and provide evidence to assess random effect using the interclass correlation coefficient (ICC). Model I was adjusted for individual-level variables, Model II with community-level factors, and Model III with variables from both individual- and community-level variables were fitted with the outcome variable.

The fixed effect is a measure of association that estimates the association between independent variables and ANC and is stated as AOR with a 95% confidence interval. The Intra-class Correlation Coefficient (ICC), Median Odds Ratio (MOR), and proportional change in variance (PCV) were computed to assess the clustering effect/variability.

## Results

### Socio-demographic characteristics of respondents

In this study, 209,538 women in 27 countries with high maternal mortality were included. Of the total, about 45.97% were aged 25–34 years and 40.77% had no formal education. The majority of study participants (86.89%) were married; however, more than a quarter (27.28%) of pregnancies were unwanted. More than half (64.20%) of the participants had media exposure and 15.11% of women had terminated their pregnancies. In our study, around 42.06% of mothers were poor and most of them (69.82%) resides in rural areas (Table 2).

**Table 2:** Socio-demographic characteristics of respondents in high maternal mortality countries.

**Variables**	**Categories**	**Unweighted frequency (%)**	**Weighted frequency (%)**
Age of mothers	15–24	63,308 (30.03)	62,993 (30.06)
	25–34	96,015 (45.54)	96,331 (45.97)
	>35	51,503 (24.43)	50,214 (23.96)
Mothers educational level	No education	89,596 (42.50)	85,420 (40.77)
	Primary education	70,029 (33.22)	70,297 (33.55)
	Secondary and above	51,201 (24.29)	53,821 (25.69)
Mothers marital status	Not in union	27,409 (13.00)	27,473 (13.11)
	Married	183,417 (87.00)	182,064 (86.89)
Wealth index	Poor	95,412 (45.26)	88,141 (42.06)
	Middle	41,477 (19.67)	41,855 (19.97)
	Rich	73,937 (35.07)	79,542 (37.96)
Media access	No	79,464 (37.77)	74,853 (35.80)
	Yes	130,914 (62.23)	134,255 (64.20)
Mothers occupation	Not working	80,897 (39.89)	79,716 (39.52)
	Working	121,882 (60.11)	121,971 (60.48)
Birth order	1	42,807 (20.30)	43,497 (20.76)
	2–5	120,777 (57.29)	120,555 (57.53)
	>6	47,242 (22.41)	45,485 (21.71)
Ever had a terminated pregnancy	No	172,949 (85.19)	171,420 (84.89)
	Yes	30,067 (14.81)	30,515 (15.11)
Pregnancy	Wanted	149,087 (73.47)	146,796 (72.72)
	Unwanted	149,087 (26.53)	55,066 (27.28)
Sex of household head	Male	167,569 (79.48)	167,160 (79.78)
	Female	43,257 (20.52)	42,378 (20.22)
Residence	Urban	60,959 (28.91)	63,243 (30.18)
	Rural	149,867 (71.09)	146,294 (69.82)
Community-level women education	Low	106,091 (50.32)	102,603 (48.97)
	High	104,735 (49.68)	106,935 (51.03)
Community-level poverty	Low	105,446 (50.02)	108,432 (51.75)
	High	105,380 (49.98)	101,106 (48.25)
Community-level media usage	Low	105,444 (50.01)	101,379 (48.38)
	High	105,382 (49.99)	108,159 (51.62)

### The pooled prevalence of ANC4+ visits in countries with high maternal mortality

**Figure 1:**
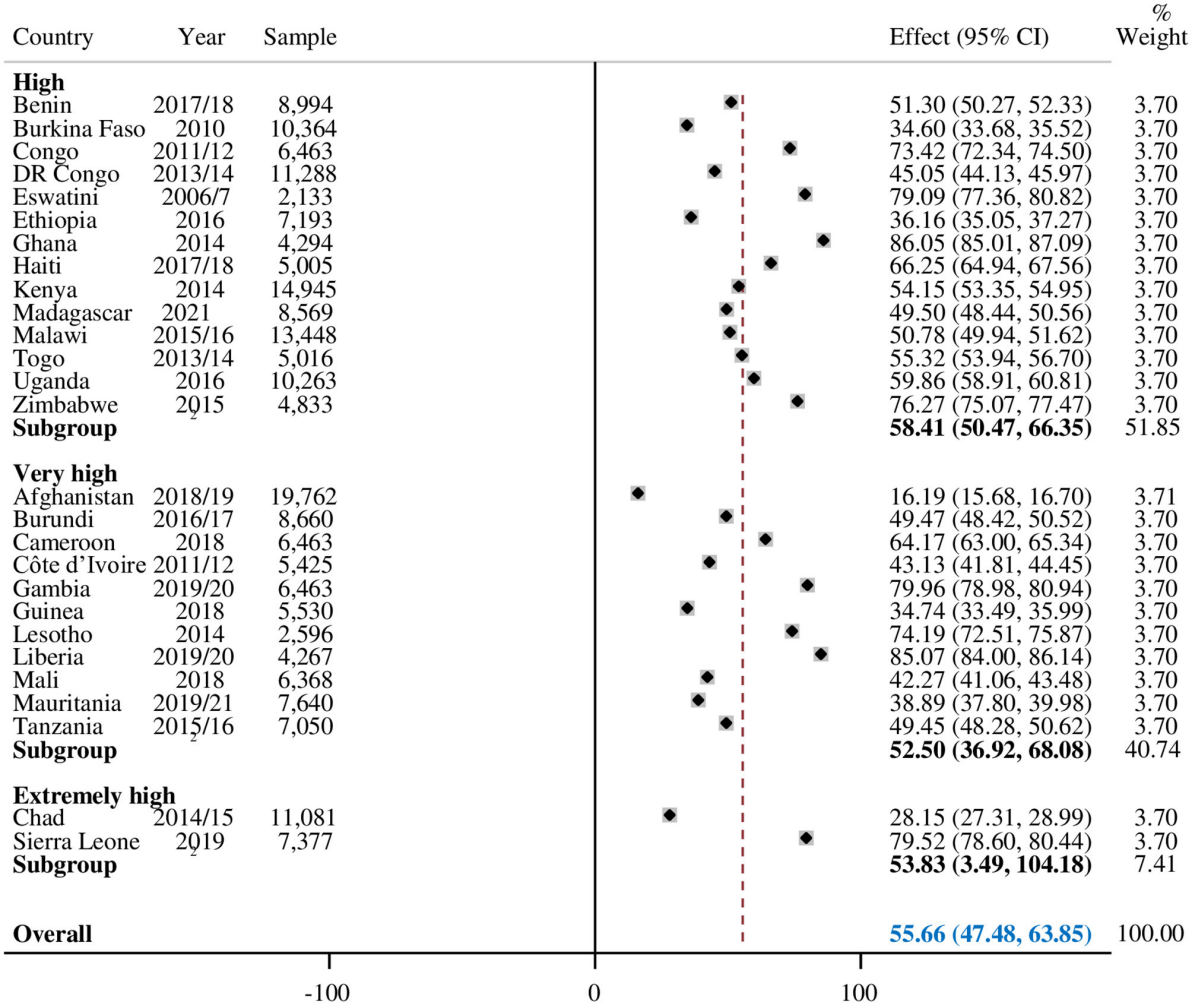
The pooled prevalence of optimal ANC in countries with high maternal mortality.

The pooled prevalence of adequate ANC visits in countries with high maternal mortality was found at 55.66% (95% CI: 47.48–63.85). Ghana (86.05%) had the highest ANC4+ visit and Liberia (85.07%) was in second place. Afghanistan was the country with the least ANC4+ visits, which was 16.19% (Figure 1).

### Multilevel logistic regression analysis of adequate ANC visits

In the multilevel analysis, mothers aged 25–34 years, mothers aged 35–49 years, maternal primary, and secondary education and above, working mothers, married, had media access, middle and rich, wanted pregnancy, had terminated pregnancy, female household head, and high community education were significantly associated variables with increased ANC visits while being rural residents and birth orders 2–5 and >5 were associated with low ANC visits (Table 3).

**Table 3:** Multivariable multilevel logistic regression analysis results of both individual-level and community-level factors associated with ANC visits in high maternal mortality countries.

**Variables**	**Categories**	**Null model**	**Model I**	**Model II**	**Model III**
			**AOR [95% CI]**	**AOR [95% CI]**	**AOR [95% CI]**
Age of mothers	15–24		1.00	—————	1.00
	25–34		1.26 (1.22–1.30)^***^	—————	1.24 (1.21–1.28)^***^
	35–49		1.55 (1.49–1.61)^***^	—————	1.52 (1.47–1.57)^***^
Mothers educational level	No education		1.00	—————	1.00
	Primary education		1.79 (1.75–1.83)^***^	—————	1.79 (1.75–1.83)^***^
	Secondary and above		3.21 (3.12–3.30)^***^	—————	3.02 (2.94–3.10)^***^
Mothers occupation	Not working		1.00	—————	1.00
	Working		1.39 (1.36–1.42)^***^	—————	1.41 (1.38–1.44)^***^
Mothers marital status	Not in union		1.00	—————	1.00
	Married		1.07 (1.03–1.10)^***^	—————	1.09 (1.06–1.13)^***^
Birth order	1		1.00	—————	1.00
	2–5		0.76 (0.74–0.79)^***^	—————	0.77 (0.74–0.79)^***^
	>5		0.55 (0.53–0.58)^***^	—————	0.56 (0.55–0.60)^***^
Media access	No		1.00	—————	1.00
	Yes		1.34 (1.31–1.38)^***^	—————	1.31 (1.28–1.33)^***^
Wealth index	Poor		1.00	—————	1.00
	Middle		1.09 (1.06–1.12)^***^	—————	1.04 (1.02–1.07)^***^
	Rich		1.34 (1.30–1.37)^***^	—————	1.08 (1.05–1.12)^***^
Pregnancy	Wanted		1.00	—————	1.00
	Unwanted		0.93 (0.91–0.95)^***^	—————	0.93 (0.91–0.95)^****^
Had terminated pregnancy	No		1.00	—————	1.00
	Yes		1.17 (1.14–1.20)^***^	—————	1.15 (1.12–1.18)^***^
Sex of household head	Male		1.00	—————	1.00
	Female		1.21 (1.18–1.25)^***^	—————	1.20 (1.17–1.23)^***^
**Community-level variables**					
Residence	Urban		—————	1.00	1.00
	Rural		—————	0.42 (0.41–0.43)^***^	0.62 (0.61–0.64)^***^
Com. women's education	Low		—————	1.00	1.00
	High		—————	1.29 (1.21–1.37)^***^	1.08 (1.01–1.57)^***^
Community poverty	Low		—————	1.00	1.00
	High		—————	1.02 (0.95–1.09)	1.00 (0.94–1.07)
Com. media usage	Low		—————	1.00	1.00
	High		—————	1.00 (0.93–1.07)	0.99 (0.87–1.06)
**Random effect**					
	Variance	0.60	0.44	0.50	0.33
	ICC	0.15	0.12	0.13	0.09
	MOR	2.01	1.71	1.83	1.48
	PCV	Reff	26.60	16.60	45.00
**Model comparison**					
	Log likelihood ratio	−100,585	−92,108	−97,815	−91,665
	Deviance	201,170	184,216	195,630	183,330

*** P-value < 0.001.

ICC, intracluster correlation coefficient; MOR, median odds ratio; PCV, proportional change in variance; AOR, adjusted odds ratio; CI, confidence interval; VIF, variance inflation factor; Com. Media, community media usage; Com. women's education, community women's education status.

## Discussion

Maternal mortality is a major public health problem, particularly in low- and middle-income countries. Early detection and intervention of complications that could happen during pregnancy have a paramount advantage and ANC visit is among those early opportunities. Optimal antenatal care is a key strategy to reduce maternal and child mortality as stated by the WHO and it could be influenced by many factors. Our study revealed a parallel relationship between women's age and ANC4+ contacts, as women's age increases, the odds of ANC4+ visits also increased. Those mothers aged 25–34 years and mothers aged 35–49 years were more likely to have adequate ANC visits compared to mothers aged 15–24 years. This finding is in agreement with previous studies conducted elsewhere (27–29). This could be because older women had more experience and better knowledge because of previous exposure to healthcare providers (30, 31).

In countries with high maternal mortality, the educational status of mothers had a similar directional relationship with ANC4+ visits. Generally, education enables women to obtain a better knowledge of the risk of pregnancy to themselves and their children. Therefore, educated mothers could have increased healthcare-seeking behavior to mitigate this risk and lead to adequate ANC usage (32–35). It has also been reported that education increased women's media penetration of the importance of ANC visits and increased the knowledge of optimal ANC usage importance (36, 37).

Occupation has been linked with ANC4+ visits (38). In our study, working mothers were more likely to have ANC4+ visits compared to their counterparts (39–41). This could be because working mother had their income and might alleviate transportation cost problems. In addition, women who generate an income might be less dependent on their husbands/partners and have better autonomy to utilize ANC frequently. However, husband/partner cooperation could increase self-esteem, reduce anxiety, and encourage the women to utilize optimal ANC visits (42–44). Husband involvement in ANC follow-up has a crucial role in pregnancy outcomes and is highly recommended by the WHO (45).

Media have been the easiest and quickest to disseminate information related to maternal health which could influence the utilization of ANC frequently. We found that women who had media exposure experienced ANC4+ compared to their counterparts (46, 47). In addition, the wealth index found a significant determinant for women to utilize adequate ANC. In our findings, the household of middle-income and the richest household were more likely to have ANC4+ visits compared to the poorest. Several previous studies were in agreement with this finding (48, 49). Although ANC services are provided for free in low- and middle-income countries, pregnant women who had financial problems may find it difficult to cover laboratory expenses and reach healthcare facilities. This could lead to delaying and inadequate ANC visit, and the impact could be high for those who live far from health institutions as the transportation cost is high.

Pregnancy wantedness has been an important determinant to utilize adequate ANC (50–52). Unwanted pregnancy could be associated with inadequate ANC usage because women might have denial and become careless about the child and their health. On the contrary, women who were pregnant because they want could have better psychosocial support from a partner, family member, relative, or friend who could lend support to the woman if any problem would arise. The introduction of life-saving modern obstetrics is difficult if the psychosocial needs of the women in these poor countries are not being taken care of. This study also revealed that women who had a history of terminated pregnancy were more likely to attend optimal ANC visits. This could be because that mothers who already experienced pregnancy termination for different reasons would be more alert to avoid such problems again and could have optimal ANC. It has been reported that women who assume that pregnancy is a risky event were more likely to attend ANC4+ visits (53).

Birth orders were found significantly associated with ANC4+ utilization. Women who had birth orders 2–5 and >5 were associated with low ANC visits compared with single birth orders. Our finding is also supported by previous studies in Ethiopia (34, 35). This might be due to increased confidence from a previous pregnancy and childbirth experience, and constraints of time and resources among women who had multiple birth orders (54). This study also revealed that place of residence was a significant factor for ANC4+ utilization. The odds of ANC4+ utilization were low among women of rural residents as compared to urban residents. This finding is in line with previous studies conducted in Nigeria (55, 56) and Ethiopia (34, 57, 58). The potential justifications for this discrepancy could be because of inequalities in healthcare service accessibility, infrastructure, and quality in service delivery in the rural and urban setups.

The strength of this study is that it used a large sample size and had adequate power to detect the true effect of the independent variables. It was based on an appropriate statistical method (multilevel analysis) to address the data's hierarchical nature. As a limitation, since the study used cross-sectional data, a causal relationship cannot be established. In addition, because it was based on the information contained in the dataset, potential variables including healthcare access, insurance, and quality healthcare are missed in the analysis.

## Conclusion

With high disparity among countries, ANC4+ utilization in countries with high mortality was low. Both individual-level factors and community-level factors were significantly associated with ANC4+ utilization. Therefore, this study revealed that policymakers, stakeholders, and health professionals should give special attention and intervene by targeting rural residents, uneducated mothers, economically poor women, and other significant factors. However, these recommendations should be considered for country-specific contextual factors, considering the different cultural orientations and varied health systems.

## Data availability statement

Permission to access the data in this study was obtained from the measure DHS program via online request. The website and the data used were publicly available with no personal identifier.

## Ethics statement

The studies involving human participants were reviewed and approved by UOG IRB. The patients/participants provided their written informed consent to participate in this study.

## Author contributions

DC: conceptualization. DC, DGB, and AZA: study design. DC, DGB, TAF, KS, MHA, SA, YYS, DMG, MD, and AZA: execution, acquisition of the data, analysis, interpretation, writing, reviewing, and editing. All authors contributed to the article and approved the submitted version.
